# Survival after Drowning with Cardiac Arrest and Mild Hypothermia

**DOI:** 10.5402/2011/895625

**Published:** 2011-04-26

**Authors:** S. S. Rudolph, S. Barnung

**Affiliations:** Department of Anaesthesia, Centre of Head and Orthopaedics, Copenhagen University Hospital, Rigshospitalet, Blegdamsvej 9, 2100 Copenhagen, Denmark

## Abstract

The current guidelines for resucitation following hypothermia and submersion with cardiac arrest state that rewarming should be continued until a core temperature of 32–34°C is achieved, after which death can be declared if no return of spontaneous circulation has occurred. As no randomized, controlled trials exist, these treatment guidelines are mostly based on a pragmatic approach. Wheater to start or stop resuscitation is notoriusly difficult. Submersion time, water temperature, and prompt resuscitation seem to be crucial factors for outcome. We report a case of successful resuscitation after the use of mechanical chest compressions and extracorporeal circulation in a patient with cardiac arrest due to submersion and accompanying mild hypothermia with a core temperature of 32,2°C caused by submersion.

## 1. Introduction

Submersion with cardiac arrest is a great challenge. Submersion time, water temperature, and prompt resuscitation seem to be crucial factors for outcome. Submersion in icy water (<5°C) may provide protection against cerebral hypoxia. Case reports have shown that survival with good neurologic outcome is possible. As no randomized, controlled trials exist, treatment guidelines are mostly based on a pragmatic approach. The current guidelines state that rewarming should be continued until a core temperature of 32–34°C is achieved [1], after which death can be declared if no return of spontaneous circulation has occurred. 

We report a case of successful resuscitation after the use of mechanical chest compressions and extracorporeal circulation in a patient with cardiac arrest due to submersion and accompanying mild hypothermia with a core temperature of 32,2°C caused by submersion.

## 2. The Case

45-year-old male was winter swimming in icy water. When trying to swim from one hole in the ice to another he got disoriented and was caught under the ice. He was brought up by rescuedivers after approximately 20 minutes. He was pulseless with an initial rhythm of asystole. Endotracheal intubation was performed on the site, and he was transported to hospital with ongoing manual chest compressions and ventilation. Epinephrine 4 mg and atropine 3 mg was administered en route to the hospital. End-tidal carbon dioxide (EtCO_2_) was between 1.6 and 6 kPa. Upon arrival at the emergency department (ED), approximately 70 minutes after the accident, he was still in cardiac arrest with asystole, and mechanical chest compressions were started (LUCAS, Jolife AB, Lund, Sweden). EtCO_2_ was 3,0 kPa. Initial bladder temperature was 32,2°C. He had a profound combined metabolic and respiratory acidosis with a pH of 6.47, S-potassium was 3,9 mMol/L. Mechanical chest-compressions were continued until extracorporeal circulation (ECC) was established approximately 110 min after the accident. Approximately 190 min after the accident a central temperature of 34°C was reached; spontaneous cardiac activity was established initially as nodal rythme and then sinus rythme. Further rewarming was withheld. A bronchoscopy preformed during ECC showed signs of pulmonary edema. ECC was discontinued and he was transferred to the intensive care unit where he received further fluid resuscitation and inotropic support. Mild hypothermia was continued for 24 hours after which he was rewarmed to normothermia. He remained stable and was gradually weaned from mechanical ventilation. 

Early during rewarming universal myoclonus was observed and treatment with levetiracetam (Keppra) was initiated. After sedation was discontinued he initially remained in a stuporous-like state of consciousness, but gradually the myoclonus decreased and he regained consciousness. Initial signs of anoxic brain damage on electroencephalography (EEG) and computed tomography (CT) of the cerebrum also regressed on repeated EEG and CT. 

On day 21 after the accident he was conscious and responded coherently to questions but some disorientation and myoclonus persisted. He was transferred to a neurorehabilitation unit where he is currently making progress.

## 3. Discussion

The treatment of cardiac arrest due to cold submersion and cardiac arrest is difficult. The questions are whether the patient suffered a cardiac arrest as a consequence of hypothermia and *then *drowned or did hypothermia occur after drowning. Even though a hypothermia victim may appear dead, resuscitation and good neurologic outcome is possible, although unusual. A general assumption is “no one is dead until warm and dead”. But how “warm” is warm? In the current guidelines a core temperature of 32–34°C is suggested. 

It is generally accepted that an S-potassium > 10 mMol/L (>12 in children) is a predictor of death [2] in patients suffering accidental hypothermia. Likewise a sustained low EtCO_2_ < 2 kPa in the face of good quality chest compressions is considered a maker of poor outcome in cardiac arrest. 

In the current case, the patient presented to the ED with asystole, a pH of 6.47, and a core temperature of 32,2°C with ongoing advanced cardiac life support for more than 50 minutes. Under normal conditions, cessation of resuscitation efforts would be considered. The decision to initiate rewarming by ECC even though the patient was above the lower temperature threshold presented in the guidelines was based on a clinical judgement. Furthermore an acceptable circulation had been maintained during transport as evidence by an EtCO_2_ between 1,6 and 6 kPa, and the S-potassium was 3,9 mmol/L. 

Survival from cardiac arrest depends on the chain of survival and if any link is inadequate, survival rates will diminish. In the current case good quality basic life support with manual chest compressions was established after initial rescue. Oxygenation and ventilation via endotracheal intubation were maintained and transport to an appropriate facility was provided. In case of accidental hypothermia and cardiac arrest, transport to a facility with EEC capablity is crucial, since ECC probably provides the best means of rewarming in this situation [3]. This should be considered even in the face of longer transportation. Good quality chest compressions, either manually or mechanically, must be maintained, during transport and no rewarming should be provided before ECC. 

In the current case the patient was marginally “too warm” according to current guidelines [1, 4], and further resuscitation efforts could have been considered futile. We chose to proceed till rewarming by ECC based on clinical judgement and on the fact that adequate cardiopulmonary resuscitation had been provided. Temperature measurements are inherently imprecise and may not reflect core temperature [5, 6], which should be remembered when caring for the hypothermic patient.
